# Do wild raccoons (*Procyon lotor*) use tools?

**DOI:** 10.1007/s10071-020-01430-y

**Published:** 2020-10-22

**Authors:** F. Blake Morton

**Affiliations:** Department of Psychology, University of Hull, Hull, HU6 7RX UK

**Keywords:** Innovation, Technical intelligence, Cognitive evolution, Culture, Physical cognition, Opportunity

## Abstract

Being able to make and use tools was once considered to be an evolutionary hallmark of our species, but has since been documented in other animals. However, for reasons that remain unclear, not all species naturally use tools. Racoons (*Procyon lotor*) are generalist carnivores that possess many of the physical, cognitive, and behavioural characteristics linked to tool use in other species (e.g. manual dexterity, tactile exploration, relatively large brains, extractive foraging, and sociality). Although raccoons have not been observed using tools outside of experimental captive conditions, wild data involving objective psychometric tests are needed. The current study administered a tool-related task to a wild population of raccoons from 20 locations within the Croatan National Forest, USA. The task required participants to use a stick to extract food from a pipe. To facilitate interpretations of their performances on the task, data were obtained on natural tool availability at the field site and participants’ mode of exploring the novel task. None of the participants solved the task despite natural sticks (suitable for solving the task) being widely available across testing locations. Participants were equally likely to smell versus handle novel sticks, which were provided at testing platforms. Limited tactile exploration, but not tool availability, could be at least one factor that reduces these raccoons’ opportunities to interact with and learn about novel tools like sticks.

**Electronic supplementary material:**

The online version of this article (10.1007/s10071-020-01430-y) contains supplementary material, which is available to authorized users.

## Introduction

Tools and other technologies have enabled humans to thrive in some of the harshest environments on the planet, explore the deepest regions of outer space, and put astronauts on the moon. Until the 1960s, scientists believed that being able to make and use inanimate objects as tools to manipulate the environment and achieve a goal was one of the defining characteristics that separated our species from other animals (Oakley 1944; Leakey 1961). Such abilities have since been documented throughout the animal kingdom, challenging notions of what it means to be a "cognitively advanced" and "technically intelligent" species (Beck 1980; Seed and Byrne 2010; Bentley-Condit and Smith 2010). However, not all species naturally use tools (Bentley-Condit and Smith 2010), suggesting that certain factors may constrain its evolution.

Raccoons are a medium-sized meso-carnivorous mammal native to North America (Gehrt 2003). They have relatively large brains and neuronal densities comparable to dogs and non-human primates (Jardim-Messeder et al. 2017). As one might expect given their relatively large brain size, they perform well on cognitive tests and show innovative and flexible behaviour (Davis 1907; Johnson and Michels 1958; Michels et al. 1961; Dalgish and Anderson 1979; Stanton et al. 2017; Daniels et al. 2019), which may facilitate their ability to colonise and thrive in a wide range of ecosystems outside their native range (e.g. Europe and Japan) (Louppe et al. 2019). Captive raccoons do not spontaneously use tools to solve tasks, but under experimental conditions, they can use inanimate objects (e.g. rocks) to manipulate their environment (e.g. raise water levels) to achieve a goal (e.g. reach food), thereby demonstrating that raccoons are capable of tool use (Stanton et al. 2017). In terms of anatomy, raccoons have excellent vision and manual dexterity for tactile exploration and object manipulation (e.g. Davis 1907; Johnson and Michels 1958; McClearn 1992; Iwaniuk and Whishaw 1999). In terms of behaviour, raccoons engage in extractive foraging (e.g. birdfeeders and clams) (Gehrt 2003; Simmons et al. 2014) and form social networks (Gehrt 2003; Hirsch et al. 2013). Thus, raccoons possess many of the physical, cognitive, and behavioural characteristics often associated with tool use in other species (e.g. van Schaik et al. 1999; Okanoya et al. 2008; Overington et al. 2009; Mann et al. 2012; Rutz and St Clair 2012; Biro et al. 2013; Sanz and Morgan 2013; Lee and Moura 2015). Although there have been no published reports of raccoons naturally or spontaneously using tools outside captive experimental conditions (Bentley-Condit and Smith 2010; Stanton et al. 2017), formal field studies involving objective psychometric tests are needed.

The current study administered a tool-related task to a wild population of raccoons in the Croatan National Forest, North Carolina, USA. Having access to materials that can be used as tools (inanimate objects) provides essential opportunities for learning about their physical properties and possible functions as tools (Fragaszy et al. 2005; Visalberghi et al. 2007; Sanz and Morgan 2013; Fujii et al. 2015). Similarly, given the tactile nature of tool use, exploring one’s environment through physical rather than alternative means (e.g. smell, vision, or taste) creates opportunities to discover and use tools (van Schaik et al. 1999). Thus, to facilitate interpretations of raccoons’ performances on the task, data were also obtained on natural tool availability at the field site as well as participants’ mode of exploring the task.

## Methods

### Study site

The Croatan National Forest, which is located on the coast of North Carolina (N34° 51.624′ W77° 03.165′), was established in 1936 as a multi-use US National Forest. It is boarded on three sides by the Neuse River, Bogue Sound, and White Oak River, respectively, and is surrounded by a mosaic of farming, commercial, and housing developments. The forest covers approximately 647 km^2^ of land and is characterised by at least seven dominant ecosystems, including long-leaf pine *(Pinus palustris*) forests, cypress (*Taxodium distichum*) swamps, pocosins, salt estuaries, open savannahs, sand beaches, and mixed pine/hardwood forests. There are four ‘wilderness areas’ that are off-limits to the public, some of which include pristine longleaf pine stands. The forest is managed by the US Forest Service of the US Department of Agriculture, which allows controlled timber extraction within designated areas. Fire is part of the natural ecology of this region and the US Forest Service conducts prescribed burns within the Croatan to reduce the hazard of uncontrollable wildfires. Other human activities allowed within the Croatan include hiking, camping, boating, biking, all-terrain vehicle driving, horse riding and seasonal hunting and fishing.

The study spanned one summer and two winter seasons between 2019 and 2020 (see “Task design and testing locations”). Total precipitation was 119.4 mm in summer and 180.1 mm in winters. Average minimum temperature was 21.7 ± 2.4 °C in summer and 5.2 ± 5.3 °C in winters. Average maximum temperature was 33.2 ± 2 °C in summer and 17 ± 5.7 °C in winters. Weather data came from the National Oceanic and Atmosphere Administration (https://ncdc.noaa.gov/cdo-web/search, retrieved 9 March 2020).

### Subjects

Aside from the current study, no formal studies have been published on the raccoon population within the Croatan. Subjects were wild, unmarked, and free ranging. Thus, the identities, sexes, and ages of raccoons that participated in cognitive testing could not be determined. Raccoons were classified as fully weaned if they consumed the hard food rewards provided at testing platforms and were larger than the length of the pipe task (30 cm) (Gehrt 2003; Okuyama et al. 2013).

### Task design

**Fig. 1:**
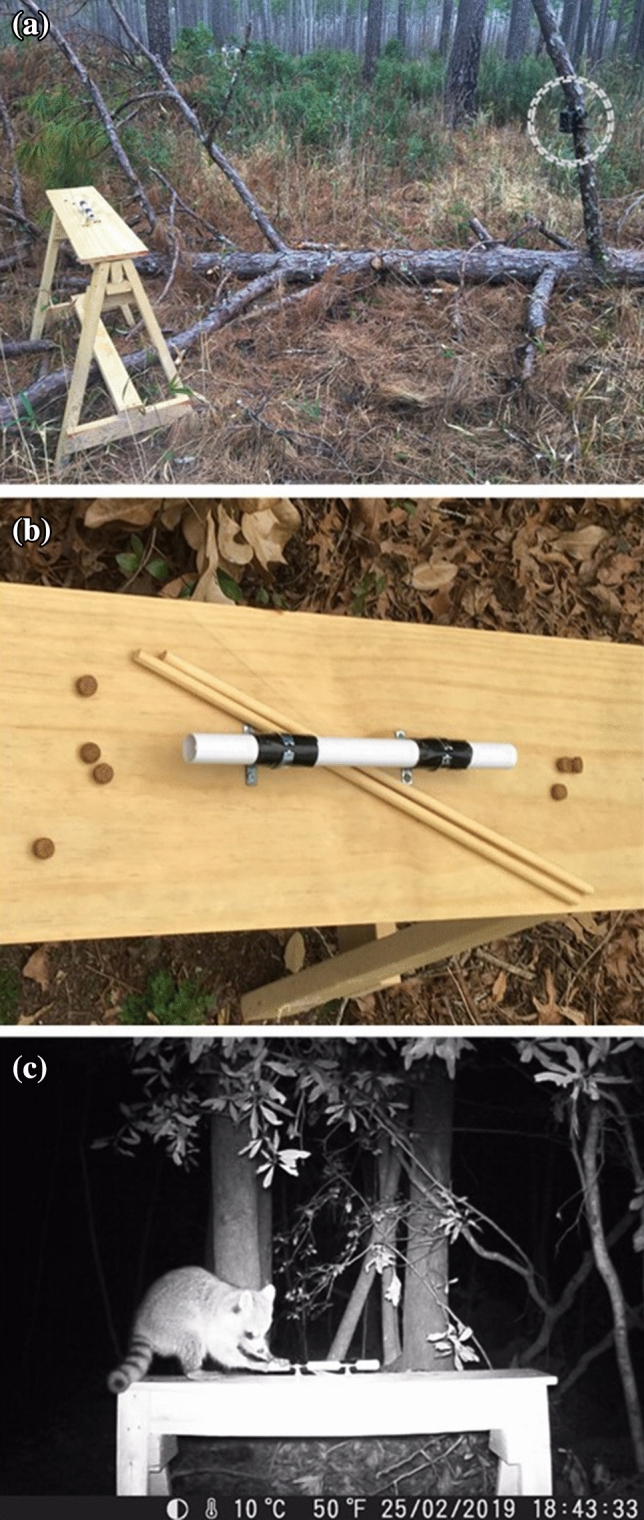
Images depicting **a** the task apparatus on a testing platform with a trail camera (circled), **b** the task setup, including two sticks and pieces of dog food (high value food rewards) located inside and outside the pipe to encourage motivation to engage, and **c** a screenshot from trail camera footage of a raccoon attempting to extract the food rewards from the pipe using their hands

The task, hereafter the “stick task”, required raccoons to use a stick to push or rake food from a pipe (Fig. 1). Many “tool kits” among wild animals include sticks for extracting out-of-reach food (van Schaik et al. 1999; Tebbich et al. 2007; Moura and Lee 2004; Rutz and Clair 2012). In the wild, animals might acquire knowledge about the properties and possible tool-related functions of sticks if, for example, they inadvertently dislodged food after displacing a stick, leading to positive reinforcement and repetition of the behaviour in the future (Alcock 1971). Stick tasks have been used to assess tool-using abilities in chimpanzees (*Pan troglodytes*), corvids, and capuchins (*Sapajus apella*) (Visalberghi et al. 1995; Bagotskaya et al. 2013), all of which could solve even more complex versions of the task used in the present study.

Approximately six pieces of commercial dog or cat food, i.e. high value bait for raccoons (Taulman and Williamson 1994; Andelt and Woolley 1996; Gehrt 2003; Schlexer 2008), were placed in the middle of a PVC pipe (length: 30.2 cm, diameter: 2 cm). The PVC material was too thick for subjects to break open and both ends of the pipe were too small for subjects to insert their hands to reach the food. The pipe was fixed to a wooden platform (length: 122 cm, width: 23.4 cm, height: 90.5 cm) using metal clamps to prevent animals from removing or tilting them (Fig. 1a, b). Raccoons are natural climbers, spend much of their time arboreally (Gehrt 2003), and were therefore capable of climbing onto the platforms. Two sticks made from smooth processed wood (length: 45.2 cm, diameter: 0.9 cm) were placed next to the pipe (Fig. 1a, b); raccoons could either use these novel sticks or natural sticks that they found themselves from their surroundings.

High value bait (e.g. dog/cat food, fish oil, and/or hot dog water) (Taulman and Williamson 1994; Andelt and Woolley 1996; Gehrt 2003; Schlexer 2008) was placed freely on each platform, outside the pipe, to motivate raccoons to approach platforms and engage in testing. Bait was either an olfactory sign of food presence, or in quantities too small to satiate the visiting racoons (e.g. 8 pieces of dried dog food). Previous research has reported no negative impact of human scent or trail cameras on wild raccoons’ willingness to approach materials manufactured and handled by humans (Munoz et al. 2014; Edmunds et al. 2018).

Raccoons should be physically and perceptually capable of operating the task. Although they are not entirely “primate-like” in terms of manual dexterity, raccoons are well-known for being able to use their hands to lift, hold, push, pull, and/or carry a wide variety of objects (e.g. locks, latches, levers, lids, plugs, rocks, strings, cups, and drawers) that vary in complexity, length, diameter, rigidity, and weight (e.g. Davis 1907; McDougall and McDougall 1931; Michels et al. 1961; Iwaniuk and Whishaw 1999; Snow et al. 2017; Stanton et al. 2017; Daniels et al. 2019). This includes being able to grab, pull, and vertically lift smooth wooden sticks of a similar size (30.5 cm) to those used in the current study (McDougall and McDougall 1931). Although indeed raccoons frequently use both hands to grasp objects (Iwaniuk and Whishaw 1999), handling and manipulating the sticks using their hands as “tongs” is all that would be necessary to solve the stick task. Davis (1907) also notes that through practice, raccoons can acquire the ability to use each forepaw independently with greater quickness and accuracy than they formerly could using both hands together. Similarly, Stanton et al. (2017) note that raccoons can grip and manoeuvre the handle of a metal scoop with one hand to insert it into a pipe.

The height of the task should also be appropriate for raccoons, since the skeletal morphology of this species includes a humerous, ulna, radius, and a wide, fan-like scapula with a subscapular fossa (Iwaniuk and Whishaw 1999). These bones enable raccoons to have extensive freedom of movement with their forelimbs (e.g. 180° vertical movement and rotation) without having to perform manual actions from a set posture or orientation (Iwaniuk and Whishaw 1999). Given that raccoons also have mouths to aid manoeuvring objects around in their hands (Davis 1907; McDougall and McDougall 1931), this gives them even more dexterity to aid manipulations/positioning of objects in ways that “handless” stick-using species cannot (e.g. birds).

In terms of visuo-motor skill, experimental studies show raccoons have adequate abilities for attending to fine-scale features of objects (Michels et al. 1961) and directing their hands towards relatively small targets (e.g. coins, peanuts, and buttons; Davies 1907; Breland and Breland 1961; Iwaniuk and Whishaw 1999). Their binocular vision also allows them to perceive depth and pick objects up and place them into containers, including pipes (Stanton et al. 2017). Thus, raccoons in the current study could—at the very least—grab one end of the stick and insert it into the pipe, then use their hand(s) and/or mouth to push the remaining segment of the stick forward until the stick is fully inside the pipe.

### Task administration

Tasks were administered once in 70 locations throughout the Croatan, which included a range of habitats (e.g. mixed hardwood forests, pine forests, swamps, and savannahs) suitable for raccoons (Gehrt 2003). Locations were spaced at least 1 km apart. None of the locations contained anthropogenic sources of food (e.g. garbage bins) accessible to raccoons.

Testing took place in one summer season (17 June–29 July 2019) and two winter seasons (13 February–1 March 2019 and 4–25 February 2020). For logistical reasons, tasks were available at 9 or 10 locations at any given time for 8.9 ± 7.4 days per location. Any animal could voluntarily participate in testing during these times, but only data from the first animal to visit a location were used in analyses.

### Recording raccoon behaviour on testing platforms

An infrared motion-sensor camera (Enkeeo PH760) was placed horizontally on a tree away from each testing platform to record subjects’ behaviour (Fig. 1a). Cameras had a 120° sensing angle, a triggering distance of 20 m, and were set to their maximum sensitivity to ensure they would detect any movement at or around the platforms. Video lengths were set to their maximum coverage, i.e. a five second trigger delay, 10-min recording bouts and 5 s in between each bout. Camera lenses were sprayed with defogger and, where necessary, understory vegetation was removed to ensure optimal visibility between the camera and platform. Raccoons that operated the task were considered to be participants; the amount of time participants spent operating the task was based on the total amount of time they spent using their hands, mouth, and/or sticks to extract the food from the pipe while they were on the platform (Fig. 1c). An independent observer randomly scored 50% of videos to perform an interobserver reliability test with the original coder (F.B.M.).

### Natural stick availability

The Croatan is characterised by a diverse range of ecosystems (see “Study Site”), but it was unclear whether or to what extent natural sticks would be readily available in some of them (e.g. treeless savannahs and fire-degraded habitats). Moreover, even if a given location contained woody debris, it was uncertain whether the right kind of debris (e.g. sticks without branches and of a certain length, diameter and straightness) would be available for raccoons to have opportunities to interact with and learn how to use them as tools. Thus, data collection on the availability of natural tools within the Croatan were evaluated.

Photos were taken in February 2019 at each of the 70 locations where the task was administered and later coded for the presence or absence of at least one natural stick that was suitable for solving the task. In February 2020, an “in-person” search for sticks was conducted at 35 (50%) of these locations. For both the photos and in-person searches, the sticks needed to be a suitable length and width for extracting food from the pipe and found with relatively little effort, which was defined in this study as any viable stick that could be found in under 10 s within a 2 m radius of the platform (Fig. 1 in ESM 1). An independent observer randomly scored 50% of photos to perform an interobserver reliability test with the original coder (FBM).

### Novel stick exploration

Trail camera videos were used to code whether raccoons explored the novel sticks by sniffing them with their noses and/or touching them. An independent observer randomly scored 50% of videos to perform an interobserver reliability test with the original coder (FBM).

### Reducing the risk of pseudoreplication

Raccoons that participated in this study could not be marked for identification and their home ranges may have overlapped (Gehrt 2003). Most home range estimates for the species fall between 0.5 and 3 km^2^ despite wide variation in geographic location, sample size, and methodology (e.g. Johnson 1970; Gehrt and Fritzell 1997; Walker and Sunquist 1997; Gehrt 2003). In the south-eastern United States where the current study took place, the largest average home range on record for a population is 0.7 km^2^ in females and 2.6 km^2^ in males (Walker and Sunquist 1997). Thus, after collecting data across all 70 locations, the risk of resampling the same individuals was reduced in the current study by only analysing videos from locations that were > 3.4 km^2^ apart, i.e., larger than the typical home range of the species plus an additional 10% “buffer”. The risk of resampling was further reduced by only using videos of the first raccoon to visit a given location from the time they first climbed onto the platform until they climbed down to the ground and moved out of the camera’s range of view. Finally, in some cases, it was possible to distinguish between raccoons based on their physical markings (see “Video Analysis”).

### Video analysis

Raccoons were recorded at 23 of the 70 locations and were all fully weaned. Two locations were < 3.4 km^2^ apart and so the video from one of these locations was randomly selected. On four other occasions, raccoons visited locations that were < 3.4 km^2^ but were included in analyses because it was possible to distinguish between individuals based on physical markings (e.g. tail band length and colouration) (Figs. 2–6 in ESM 1). Due to camera malfunctions, videos from two locations only depicted raccoons as they climbed down from the platforms. Thus, videos from 20 locations (10 from summer and winter, respectively) were retained for analyses of raccoons’ participation and performance on the task. Lastly, video from one location depicted a raccoon whose body blocked their responses to the sticks. Thus, videos from 19 locations (10 from summer and 9 from winter) were retained for the stick exploration analysis.

### Statistical analyses

Two types of intraclass correlation coefficients (Shrout and Fleiss 1979) were calculated to determine interobserver agreement between raccoons’ operation time on the task (i.e. time spent trying to gain access to food in pipes). The first intraclass correlation coefficient, ICC (3, 1), indicates the reliability of single ratings. The second, ICC (3, *k*), indicates the reliability of the mean scores across *k* raters (two raters in the present study). Cohen’s kappa coefficients were calculated to determine interobserver agreement on raccoons’ methods of stick exploration (sniff, handle, or both), tool availability judged from photos (i.e. presence or absence of sticks), and the degree of overlap between the tool availability scores derived from the original photos and the in-person searches.

Chi-squared tests were used to test whether there were more locations with natural sticks available than locations where they were absent. Chi-squared tests were also used to test whether raccoons at each location were more likely to acknowledge versus ignore trail cameras, and whether they were more likely to use tactile versus alternative means (e.g. sniff) to explore the novel sticks.

A Mann–Whitney *U* test was used to compare seasonal differences (winter versus summer) in the average amount of time raccoons spent operating the task, and whether operating time differed according to whether or not the subject looked in the direction of the trail camera during their session.

The data analysed in this study are provided in Tables 1–5 in ESM 1. Statistical analyses were conducted using SPSS Version 26.

## Results

### Raccoon task participation and performance

Of the 20 locations retained for analysis, all raccoons readily approached the task up to one-fourth or less of their body length during their session. Raccoons from 17 (85%) locations acknowledged the food in pipes, for example by sniffing the open ends of the pipe. Raccoons from 16 (80%) locations (6 from summer and 10 from winter) tried to extract the food in the pipe using their teeth and/or hands. Raccoons were significantly more likely than not to engage in testing, *χ*^2^(1, *N* = 20) = 7.2, *p* = 0.0073, and were equally likely to interact with the task in winter versus summer sessions, *χ*^2^(1, *N* = 20) = 0.25, *p* = 0.617. Raccoons from 8 (40%) locations looked in the direction of the camera but immediately relaxed and resumed their activities on the platforms. Raccoons were equally likely to ignore cameras versus look in their direction, *χ*^2^ (1, *N* = 20) = 0.8, *p* = 0.371. No other animals, including other raccoons, were observed on or around the platforms during these sessions.

There was strong interobserver reliability for raccoon operation times, ICC (3, 1) = 0.972 and ICC (3, *k*) = 0.986. Raccoons’ median operating time on the task was 20.2 s. Operation time did not significantly differ between winter (Mdn = 17.13 s) versus summer (Mdn = 20.36 s), *U* = 27, *p* = 0.745, or between individuals that appeared to acknowledge (Mdn = 19.6 s) versus ignore the cameras (Mdn = 20.9 s), *U* = 31, *p* = 0.958. None of the raccoons that tried to access the rewards within the pipe were successful, and none of them attempted to use sticks to solve the task (see video in ESM 2).

### Natural stick availability

Based on the photos, natural sticks were found in under 10 s at 53 (75.7%) of the 70 locations where the task was administered. There were significantly more locations with natural sticks present than absent, *χ*^2^ (1, *N* = 70) = 18.5, *p* < 0.001. There was perfect agreement between the original scores and the independent observer’s scores, *κ* = 1, *n* = 35 locations.

Of the 35 locations where photos and in-person data were available from the same place, there was overall poor agreement between scores, *κ* < 0. In all cases of disagreement, the in-person searches revealed sticks that were not detected in photos. Natural sticks were available at all locations visited by raccoons.

### Novel stick exploration

Of the 19 locations retained for analysis, raccoons from 17 (89.5%) locations explored the sticks. Raccoons from two of these locations did not acknowledge the food in pipes. Overall, raccoons were more likely to explore versus ignore the sticks, *χ*^2^ (1, *N* = 19) = 11.84, *p* = 0.006.

There was perfect interobserver agreement for raccoon exploratory behaviour, *κ* = 1, *n* = 10 locations. Of the raccoons that explored the sticks, raccoons from 11 (64.7%) locations sniffed the sticks without handling them, while raccoons from the remaining locations sniffed and handled them (e.g. picking them up and biting/rolling them with their hands). Raccoons were equally likely to sniff versus sniff and handle the sticks, *χ*^2^ (1, *N* = 17) = 1.47, *p* = 0.225. Raccoons that only sniffed the sticks were equally likely to avoid versus indirectly touch the sticks, for instance, by stepping on them as they explored the platforms, *χ*^2^ (1, *N* = 11) = 0.818, *p* = 0.366.

## Discussion

This study investigated the tool-using abilities of wild raccoons by administering a stick-related tool task to a population within the Croatan National Forest, USA. Raccoons from 20 locations participated in testing. Although natural and experimental sticks were readily available to participants, none of the participants solved the task and individuals were no more likely to explore the tools physically versus olfactorily.

### Tool availability

Comparing photos and in-person searches of natural sticks across testing locations revealed low agreement between scores. In all cases of disagreement, however, in-person searches revealed sticks that were not detected in photos, indicating that stick abundance was likely higher than what was estimated from the photos, and may have been present at 76 to 100% of locations. Along with natural tools being present at many locations, novel sticks were provided at platforms, yet raccoons still did not attempt to use them to access food rewards. Thus, a lack of opportunities to encounter and use sticks as tools cannot explain why raccoons in this study did not use them to access the food.

Further research might investigate whether goal availability is a more crucial limiting factor for tool use in raccoons. Indeed, tool use among wild chimpanzees and sea otters (*Enhydra lutris*), for example, can depend on extractive food availability, and hence, opportunities to use tools to gain access to those food items (Sanz and Morgan 2013; Fujii et al. 2015). Further research might also consider whether the combined availability of tools and goals is more important than either one is separately. For instance, Stanton et al. (2017) found that captive raccoons could only solve a tool-related task (i.e. dropping a rock into a pipe to displace water to gain access to a reward) after researchers positioned the rock at the edge of the pipe, thereby enabling subjects to accidentally “discover” the solution by displacing it while attempting to obtain the reward with their hand. The rate of learning for any species is, of course, strongly influenced by reinforcement schedule (Bitterman and Schoel 1970). Therefore, natural sticks may not occur in close enough proximity to food-related goals to allow raccoons opportunities to discover the benefits of using sticks to gain access to food items. To test this hypothesis, one could determine whether the likelihood of raccoons using sticks as tools varies as a function of tool and goal availability (e.g. embedded food items) in the environment, and more importantly, how often wild raccoons accidentally displace natural sticks while trying to gain access to those goals.

### Novel tool exploration

Although, as previously noted, raccoons use their hands to explore and manipulate their environment (Davis 1907; McClearn 1992; Iwaniuk and Whishaw 1999; Daniels et al. 2019), like many carnivorous species (Gittleman 1991), they also possess a keen sense of smell (e.g. Burke et al. 2005; Buzuleciu et al. 2016). Thus, raccoons can use their nose to gather information about novel objects without having to handle the objects directly. In the current study, the observation that raccoons were equally likely to explore the novel tools through smell versus touch is in stark contrast to the behaviour of prolific tool users, such as humans and chimpanzees, which are much more tactically exploratory with their hands (Bjorklund and Gardiner 2011; Koops et al. 2015). In mice (*Mus musculus*), scent-impaired individuals are more physically exploratory than intact individuals (Kudyakova et al. 2007). Thus, although raccoons possess the necessary physical morphology for tactile exploration of tools, their capacity for using olfaction to explore their environment may reduce opportunities for learning about the physical properties and functions of tools. Future research on wild raccoons could test this “olfaction hypothesis” using chemical intranasal irrigation to temporarily induce loss of smell (Mast et al. 2019) which may improve raccoons' willingness to physically explore and learn about tools. Future models of species variation in tool use should also clarify whether tactile exploration is the primary mode of exploration for a species, and in particular, whether the species is more likely to use physical versus other modes of exploration like olfaction when they first encounter novel tools. This may help explain why some species are capable of using tools but rarely do so in the wild, such as bears (*Ursus arctos*) (Deecke 2012), which have a keen sense of smell.

### Dispositional versus situational effects on task performance

Studies on a range of species, including captive raccoons (Daniels et al. 2019), have found significant effects of personality on task performance (Auersperg et al. 2011; Benson-Amram et al. 2013; Damerius et al. 2017). In the current study, all raccoons readily approached the pipe and many of them willingly engaged in testing. Most raccoons also touched (either directly or indirectly) the novel sticks that were placed on platforms. Such patterns of behaviour contrast wild orangutans (*Pongo *spp*.*), which are prolific tool users despite experimental studies showing that they can take months to touch novel objects (Forss et al. 2015). It is therefore unlikely that neophobia or a lack of curiosity to approach and touch the task underpinned raccoons’ inability to solve it. However, further work might test whether dispositional effects played a role in raccoons’ willing to persist in the task (Daniels et al. 2019). Indeed, the amount of time raccoons spent operating the stick task was surprisingly brief, suggesting that while participants were initially willing to engage, they quickly lost motivation.

Demonstrating an effect from personality, however, also requires being able to rule out situational effects. For example, raccoons’ lack of persistence may have instead been due to a lack of hunger or leaving to find food elsewhere once they realised that the food rewards were not easily accessible. Grund et al. (2019) found that operation times among wild chimpanzees were similarly brief on a tool-related stick task (18.8 ± 19 s), which was linked to prior feeding and travel time, and therefore, presumably their degree of hunger. In the current study, there were no seasonal effects on racoons’ willingness to participate in the stick task nor the amount of time they spent operating it. Thus, situational effects related to seasonal differences in food abundance, and therefore variation in motivation due to hunger, are unlikely to explain why raccoons lost interest so quickly. Alternatively, raccoons’ performance on the task may reflect a year-round optimal foraging strategy in this species, whereby individuals move on to find alternative resources if their initial attempts at accessing the food are not profitable or necessary. King et al. (1974) found, for example, that a raccoon would become agitated when tested on a fixed ratio schedule compared to a fixed interval (and hence more profitable) schedule. Thus, to rule out situational effects related to profitability and/or necessity, future research might record raccoons’ activities prior to testing, by either fitting cameras to GPS collars or habituating and following them in person, which would help rule out the effects of hunger on their tool-related task performance. Further research might also increase the size and quality of food rewards used in testing, which could motivate raccoons to persist longer in trying to solve the task. Finally, future research might test whether raccoons are more likely to engage and solve tool-related tasks when alternative resources are limited throughout the year (e.g. heavily degraded habitats), which may make tool use more profitable, and perhaps even necessary, for meeting energy demands (Sanz and Morgan 2013).

### Task design

Although raccoons from the Croatan did not solve the stick task, further studies involving more raccoon populations are needed. Such work should ideally include a much larger battery of tool-related tasks.

Previous work in captive raccoons has found no relationship between measures of inhibition and innovation (Daniels et al. 2019), and the fact that raccoons can learn to use tools illustrates that they have enough inhibitory control to perform the behaviour (Stanton et al. 2017). Nevertheless, other studies have reported occasions where raccoons have struggled with inhibiting ineffectual behaviour, such as resisting the release of objects from their grasp while trying to insert them into containers (Breland and Breland 1961). Thus, future research might consider using a task that requires the participant to rake or scoop an out-of-reach reward, which is something that raccoons can learn to do (Stanton et al. 2017) and is easier for tool-using species like great apes to operate compared to tasks that require them to push food away before gaining access (Mulcahy and Call 2006; Martin-Ordas et al. 2008). Although natural and experimental sticks in the current study could be used to rake food towards the operator (pers. obs.), a different task could nevertheless be administered so that natural sticks with branches, or experimental sticks with hooks or prongs, are needed to solve it. A design of this nature may even improve the profitability of using tools to forage (St Clair et al. 2018), and in turn, increase the likelihood of raccoons being willing to operate such tools during testing. Finally, in areas where stones are part of raccoons’ natural landscape (e.g. mountainous areas), studies might also consider using the same or similar task used by Stanton et al. (2017).

## Conclusions

Although raccoons possess many of the physical, cognitive, and behavioural traits characteristic of tool-using species, the current study found that a population of wild raccoons did not solve a stick-related tool task. Limited tactile exploration, but not tool availability, could be at least one factor that reduces these raccoons’ opportunities to interact with and learn about the tool-related functions of objects like sticks.

## Electronic supplementary material

Below is the link to the electronic supplementary material.

Supplementary file1 (DOCX 2069 kb)

Supplementary file2 (MP4 72030 kb)

## Data Availability

Data analysed in this study are provided in Tables 1–5 in ESM 1.
