# Supplementary material for: Do wild raccoons (Procyon lotor) use tools?
Source: Anim Cogn. 2020 Oct 22;24(3):433–41. doi: 10.1007/s10071-020-01430-y (PMC8128817; doi:10.1007/s10071-020-01430-y)
Supplement: Supplementary file 1 — Supplementary file1 (DOCX 2069 kb) [file 10071_2020_1430_MOESM1_ESM.docx]

**Electronic Supplementary Materials**


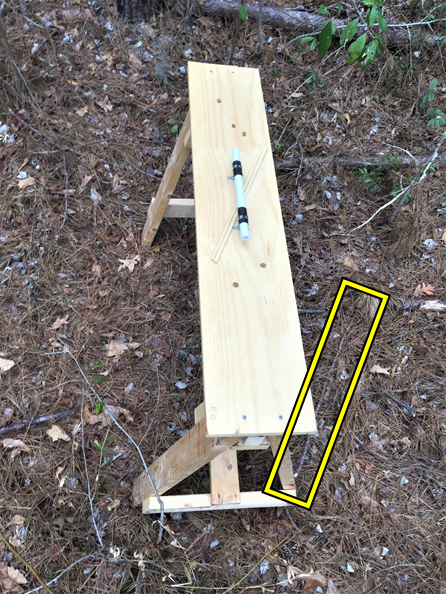


**Fig. 1** Example of a natural stick (yellow rectangle) within a 2m radius from the testing platform that could be found within 10 seconds and feasibly used as a tool to solve the pipe task

(**a**)
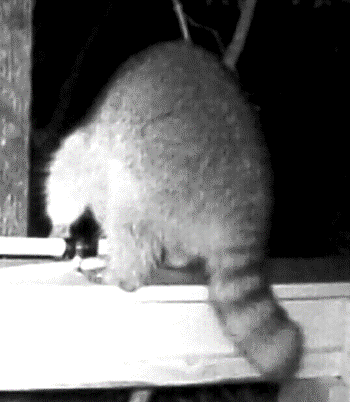
 (**b**)
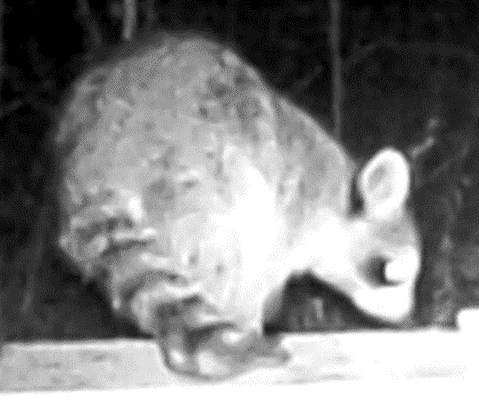


**Fig. 2** Raccoons from Zone 18 and 19 could be distinguished based on the number of black rings on their tail, which was 6 rings for the raccoon in (**a**) and 7 rings for the raccoon in (**b**). They could also be distinguished by the amount of black hair at the tip of their tails, which was far more extensive for the raccoon in (**a**) compared to the raccoon in (**b**)

(**a**)
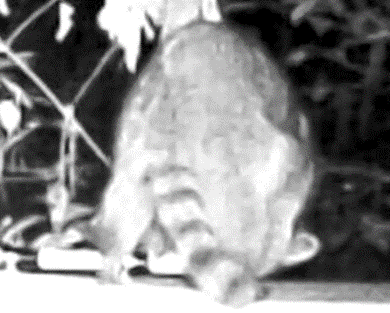
 (**b**)
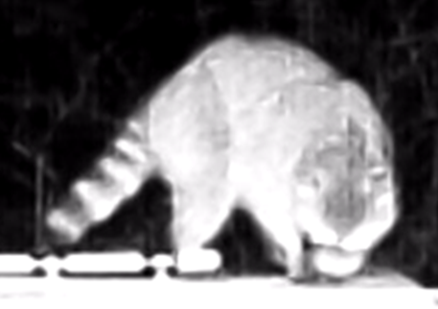


**Fig. 3** Raccoons from Zone 21 and 22 could be distinguished based on the amount of black hair at the tip of their tails, which was far more extensive for the raccoon in (**b**) compared to the raccoon in (**a**)

(**a**)
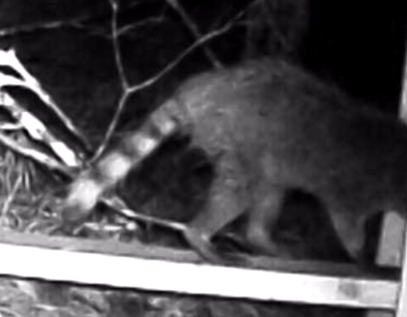
 (**b**)
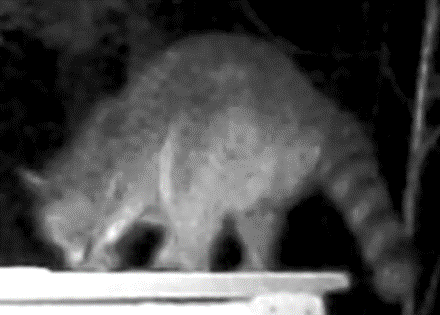


**Fig. 4** Raccoons from Zone 33 and 34 could be distinguished based on the amount of black hair at the tip of their tails, which was present on the raccoon in (**a**) but absent on the raccoon in (**b**)

(**a**)
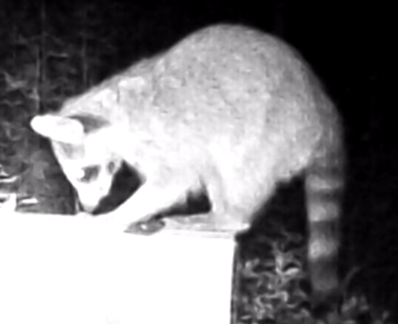
 (**b**)
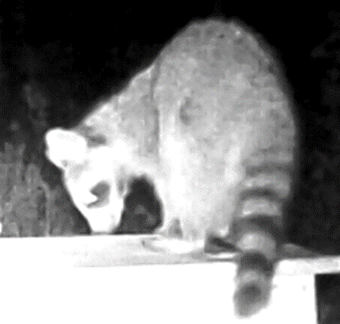


**Fig. 5** Raccoons from Zone 47 (**a**) and Zone 48 (**b**) could not be distinguished due to strong similarities in physical traits (e.g. tail length, and tail/eye patch colouration)

(**a**)
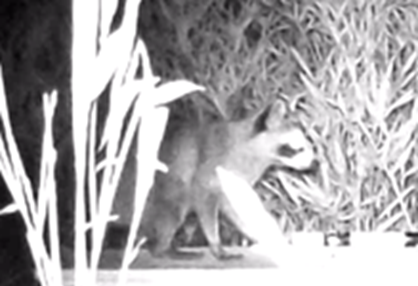
 (**b**)
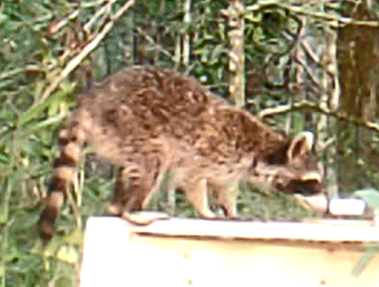


**Fig. 6** Raccoons from Zone 57 and 58 could be distinguished based on the amount of black hair around their eyes, which extended to the lower back jawline of the raccoon in (**b**) but not the raccoon in (**a**)

**Table 1** List of all locations visited by raccoons and their corresponding behaviour while on testing platforms

| Zone |  | Season | Ate free bait? | Approached pipe? | Acknowledged food in pipe? | Tried to solve? | Task operating time (s) | Looked at camera? |
| --- | --- | --- | --- | --- | --- | --- | --- | --- |
| 4 |  | Winter | Yes | Yes | Yes | Yes | 21.31 | No |
| 6 |  | Winter | Yes | Yes | Yes | Yes | 6.7 | Yes |
| 9 |  | Winter | Yes | Yes | Yes | Yes | 20.85 | No |
| 10 |  | Summer | Yes | Yes | Yes | No | ---- | No |
| 12 |  | Winter | Yes | Yes | Yes | Yes | 13.26 | No |
| 17 |  | Summer | Yes | Yes | Yes | Yes | 81.28 | Yes |
| 18 |  | Winter | Yes | Yes | Yes | Yes | 57.22 | No |
| 19 |  | Winter | Yes | Yes | Yes | Yes | 1.16 | No |
| 20 |  | Summer | Yes | Yes | No | No | ---- | Yes |
| 21 |  | Winter | Yes | Yes | Yes | Yes | 58.25 | No |
| 22 |  | Winter | Yes | Yes | Yes | Yes | 3.86 | No |
| 27 |  | Winter | Yes | Yes | Yes | Yes | 111.71 | No |
| 29 |  | Winter | Yes | Yes | Yes | Yes | 13.4 | Yes |
| 33 |  | Summer | Yes | Yes | No | No | ---- | No |
| 34 |  | Summer | Yes | ---- | ---- | ---- | ---- | ---- |
| 35 |  | Summer | Yes | Yes | Yes | Yes | 19.55 | Yes |
| 47 |  | Summer | Yes | Yes | Yes | Yes | 10.25 | Yes |
| 54 |  | Summer | Yes | Yes | Yes | Yes | 24.52 | Yes |
| 57 |  | Summer | Yes | Yes | No | No | ---- | No |
| 58 |  | Summer | Yes | Yes | Yes | Yes | 6.8 | No |
| 65 |  | Summer | Yes | ---- | ---- | ---- | ---- | ---- |
| 66 |  | Summer | Yes | Yes | Yes | Yes | 21.16 | Yes |

**Table 2** List of all locations visited by raccoons and their corresponding responses to the novel sticks provided on testing platforms

| Zone | Season | Explored sticks? | Method of stick exploration | Indirectly touched stick? (for raccoons that only sniffed) |
| --- | --- | --- | --- | --- |
| 4 | Winter | Yes | Sniff | Yes |
| 6 | Winter | Yes | Sniff | No |
| 9 | Winter | Yes | Sniff | Yes |
| 10 | Summer | Yes | Sniff | Yes |
| 12 | Winter | Yes | Sniff | Yes |
| 17 | Summer | Yes | Sniff + Handle | ---- |
| 18 | Winter | ---- | ---- | ---- |
| 19 | Winter | Yes | Sniff | Yes |
| 20 | Summer | Yes | Sniff + Handle | ---- |
| 21 | Winter | Yes | Sniff + Handle | ---- |
| 22 | Winter | Yes | Sniff | No |
| 27 | Winter | Yes | Sniff | Yes |
| 29 | Winter | Yes | Sniff | No |
| 33 | Summer | No | ---- | ---- |
| 34 | Summer | ---- | ---- | ---- |
| 35 | Summer | Yes | Sniff | No |
| 47 | Summer | Yes | Sniff + Handle | ---- |
| 54 | Summer | Yes | Sniff + Handle | ---- |
| 57 | Summer | No | ---- | ---- |
| 58 | Summer | Yes | Sniff | Yes |
| 65 | Summer | ---- | ---- | ---- |
| 66 | Summer | Yes | Sniff + Handle | ---- |

**Table 3** List of all locations where a raccoon was the first visitor to the platform

| Zone | Season | 1^st^ Visitor | Ate free bait? |
| --- | --- | --- | --- |
| 4 | Winter | Raccoon | Yes |
| 6 | Winter | Raccoon | Yes |
| 9 | Winter | Raccoon | Yes |
| 10 | Summer | Raccoon | Yes |
| 12 | Winter | Raccoon | Yes |
| 17 | Summer | Raccoon | Yes |
| 18 | Winter | Raccoon | Yes |
| 19 | Winter | Raccoon | Yes |
| 20 | Summer | Raccoon | Yes |
| 21 | Winter | Raccoon | Yes |
| 22 | Winter | Raccoon | Yes |
| 27 | Winter | Raccoon | Yes |
| 29 | Winter | Raccoon | Yes |
| 33 | Summer | Raccoon | Yes |
| 34 | Summer | Raccoon | Yes |
| 35 | Summer | Raccoon | Yes |
| 47 | Summer | Raccoon | Yes |
| 54 | Summer | Raccoon | Yes |
| 57 | Summer | Raccoon | Yes |
| 58 | Summer | Raccoon | Yes |
| 65 | Summer | Raccoon | Yes |
| 66 | Summer | Raccoon | Yes |

**Table 4** The amount of time in seconds that raccoons spent operating the task, coded by two

independent observers

|  | Coded operating times (s) | |
| --- | --- | --- |
| Zone | 1^st^ Observer | 2^nd^ Observer |
| 4 | 17.82 | 21.31 |
| 6 | 5.95 | 6.7 |
| 29 | 12 | 13.4 |
| 58 | 7.01 | 6.8 |
| 19 | 1 | 1.16 |
| 12 | 9.19 | 13.26 |
| 66 | 20.4 | 21.16 |
| 47 | 8.45 | 10.25 |
| 9 | 18.18 | 20.85 |
| 35 | 20.65 | 19.55 |

**Table 5** Natural stick availability (1=present, 0=absent) at all 70 locations based on photos coded by two independent observers and in-person searches

| Zone | Photos | | In-person search |
| --- | --- | --- | --- |
|  | 1^st^ Observer | 2^nd^ Observer |  |
| 1 | 1 | 1 | 1 |
| 2 | 1 | 1 | 1 |
| 3 | 1 | 1 | 1 |
| 4 | 1 | 1 | 1 |
| 5 | 1 | 1 | ---- |
| 6 | 1 | 1 | 1 |
| 7 | 1 | 1 | 1 |
| 8 | 1 | 1 | ---- |
| 9 | 0 | 0 | 1 |
| 10 | 1 | 1 | 1 |
| 11 | 0 | 0 | 1 |
| 12 | 1 | 1 | 1 |
| 13 | 1 | 1 | 1 |
| 14 | 1 | 1 | ---- |
| 15 | 1 | 1 | 1 |
| 16 | 1 | 1 | 1 |
| 17 | 1 | 1 | 1 |
| 18 | 1 | 1 | 1 |
| 19 | 1 | 1 | 1 |
| 20 | 0 | 0 | 1 |
| 21 | 0 | 0 | 1 |
| 22 | 1 | 1 | 1 |
| 23 | 1 | 1 | ---- |
| 24 | 1 | 1 | ---- |
| 25 | 1 | 1 | ---- |
| 26 | 1 | 1 | 1 |
| 27 | 1 | 1 | 1 |
| 28 | 1 | 1 | 1 |
| 29 | 1 | 1 | 1 |
| 30 | 1 | 1 | ---- |
| 31 | 1 | 1 | 1 |
| 32 | 1 | 1 | ---- |
| 33 | 1 | 1 | 1 |
| 34 | 1 | ---- | 1 |
| 35 | 0 | ---- | 1 |
| 36 | 1 | ---- | ---- |
| 37 | 0 | ---- | ---- |
| 38 | 0 | ---- | ---- |
| 39 | 1 | ---- | ---- |
| 40 | 1 | ---- | ---- |
| 41 | 0 | ---- | ---- |
| 42 | 0 | ---- | ---- |
| 43 | 1 | ---- | ---- |
| 44 | 0 | ---- | ---- |
| 45 | 0 | ---- | ---- |
| 46 | 1 | ---- | ---- |
| 47 | 1 | ---- | ---- |
| 48 | 0 | ---- | ---- |
| 49 | 0 | ---- | ---- |
| 50 | 1 | ---- | ---- |
| 51 | 1 | ---- | ---- |
| 52 | 1 | ---- | ---- |
| 53 | 1 | ---- | ---- |
| 54 | 1 | ---- | ---- |
| 55 | 1 | ---- | 1 |
| 56 | 1 | ---- | 1 |
| 57 | 1 | ---- | ---- |
| 58 | 1 | ---- | 1 |
| 59 | 0 | ---- | 1 |
| 60 | 1 | ---- | ---- |
| 61 | 1 | ---- | ---- |
| 62 | 0 | ---- | 1 |
| 63 | 1 | 1 | ---- |
| 64 | 0 | ---- | 1 |
| 65 | 1 | ---- | 1 |
| 66 | 1 | ---- | 1 |
| 67 | 0 | ---- | ---- |
| 68 | 1 | ---- | ---- |
| 69 | 1 | 1 | ---- |
| 70 | 1 | ---- | ---- |
